# Comprehensive Overview of Broadly Neutralizing Antibodies against SARS-CoV-2 Variants

**DOI:** 10.3390/v16060900

**Published:** 2024-06-01

**Authors:** Lingyan Cui, Tingting Li, Wenhui Xue, Sibo Zhang, Hong Wang, Hongjing Liu, Ying Gu, Ningshao Xia, Shaowei Li

**Affiliations:** 1 State Key Laboratory of Vaccines for Infectious Diseases, Xiang An Biomedicine Laboratory, School of Public Health, School of Life Sciences, Xiamen University, Xiamen 361102, Chinansxia@xmu.edu.cn (N.X.); 2 State Key Laboratory of Molecular Vaccinology and Molecular Diagnostics, Collaborative Innovation Center of Biologic Products, National Innovation Platform for Industry-Education Integration in Vaccine Research, The Research Unit of Frontier Technology of Structural Vaccinology of Chinese Academy of Medical Sciences, National Institute of Diagnostics and Vaccine Development in Infectious Diseases, Xiamen University, Xiamen 361102, China

**Keywords:** SARS-CoV-2 variants, mutations, broadly neutralizing antibodies, epitopes

## Abstract

Currently, SARS-CoV-2 has evolved into various variants, including the numerous highly mutated Omicron sub-lineages, significantly increasing immune evasion ability. The development raises concerns about the possibly diminished effectiveness of available vaccines and antibody-based therapeutics. Here, we describe those representative categories of broadly neutralizing antibodies (bnAbs) that retain prominent effectiveness against emerging variants including Omicron sub-lineages. The molecular characteristics, epitope conservation, and resistance mechanisms of these antibodies are further detailed, aiming to offer suggestion or direction for the development of therapeutic antibodies, and facilitate the design of vaccines with broad-spectrum potential.

## 1. Introduction

On 30 January 2020, the World Health Organization (WHO) declared the severe acute respiratory syndrome coronavirus 2 (SARS-CoV-2) epidemic as a public health emergency of international concern. Subsequently, on 11 February 2020, the International Committee on Taxonomy of Viruses (ICTV) named the virus as SARS-CoV-2 [1], and the WHO officially designated the outbreak of coronavirus disease as Coronavirus Disease-2019, commonly known as COVID-19 [2]. As of May, 2024, the WHO has reports over 775 million infections and 7 million deaths globally (https://covid19.who.int/, accessed on 12 May 2024).

**Figure 1:**
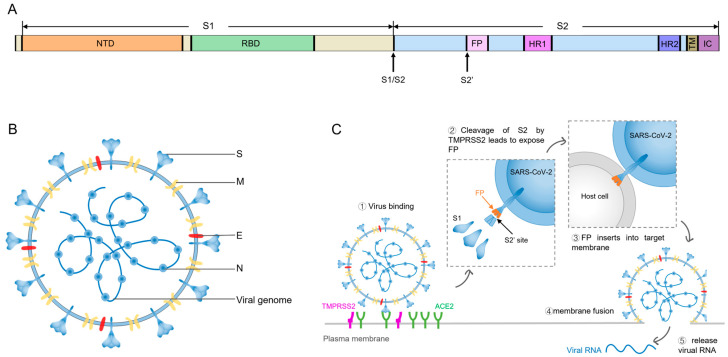
(**A**) Schematic representation of SARS-CoV-2 spike protein, highlighting key domains including N-terminal domain (NTD), receptor-binding domain (RBD), fusion peptide (FP), heptad repeat 1 (HR1), heptad repeat 2 (HR2), transmembrane domain (TM), intracellular domain (IC). (**B**) Structure of SARS-CoV-2 virus. S, spike protein; M, membrane protein; E, envelope protein; N, nucleocapsid protein. (**C**) The process of viral entry mediated by the SARS-CoV-2 spike protein.

The genome analysis reveals that SARS-CoV-2 is a novel beta-coronavirus [3,4]. The viral genome consists of an about 30 kb, positive-sense, single-stranded RNA, encoding nonstructural proteins (NSPs), accessory proteins, and structural proteins. The 16 NSPs (nsp1–nsp16) are required for viral RNA synthesis. Nine accessory proteins provide a selective advantage in the infected host. The four structural proteins, spike protein (S), membrane protein (M), envelope protein (E), and nucleocapsid protein (N), are necessary for assembly and production of the whole viral particles [5] (Figure 1B). Among them, the S protein is a class I fusion transmembrane protein, mediating virus attachment to and fusion with the host cell membrane [6]. The M protein serves as the primary structural protein in the envelope, playing a vital role in virus assembly, budding, envelope formation, and pathogenesis [7,8]. The E protein is a minor structural protein, forming pentameric protein-lipid channels within the host cell membrane, crucial for intracellular trafficking [8,9]. The N protein interacts with the RNA genome, providing protection for the RNA and facilitating the processes of RNA replication and transcription [8,10].

**Figure 2:**
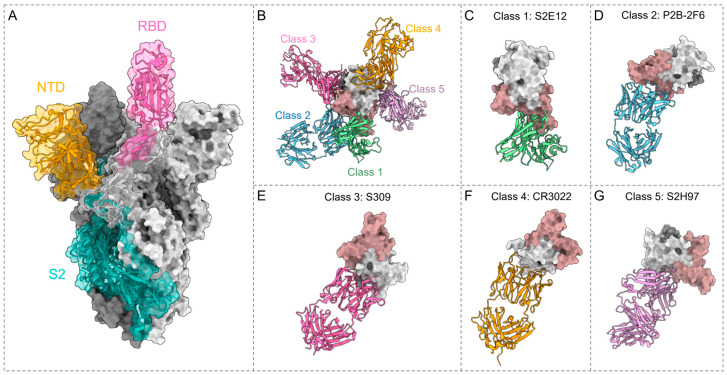
Spike structure and epitopes of class 1–5 RBD antibodies. (**A**) The spike trimer (PDB: 7DX1) is shown using a bimodal representation. Two of the protomers are displayed with their molecular surfaces in dark and grey, the third protomer is illustrated as a ribbon. (**B**) Overlay of RBD structures (light grey) alongside Class 1–5 representative antibodies, S2E12(PDB: 7R6X), P2B-2F6(PDB: 8DCC), S309(PDB: 7R6X), CR3022(PDB: 8FAH), S2H97(PDB: 7M7W), showcasing distinct binding orientations. The receptor-binding motif (RBM) is highlighted in rosy brown. (**C**–**G**) The localization of class 1–5 representative antibodies on the epitopes within the RBD.

The S protein exists as a homotrimer, comprising three protomers (Figure 2A). Each protomer contains S1 and S2 subunits, with furin cleavage sites (PRRAR) at the junction of S1/S2 subunits [11]. The furin proprotein convertase cleaves the S protein into S1 and S2 subunits in the infected cells, which greatly enhances the efficiency of viral entry [6]. The distally located S1 subunit comprises two domains, the N-terminal domain (NTD) and C-terminal domain (CTD) functioning as a receptor-binding domain (RBD) [12,13]. The S2 is a membrane-anchored subunit that consists of a fusion peptide (FP), a heptad repeat 1 (HR1), a heptad repeat 2 (HR2), a transmembrane region (TM), and an intracellular domain (IC) [14] (Figure 1A). The RBDs are located at the apex of the S trimer, adopting either the receptor-accessible “up” conformation or the receptor-inaccessible “down” conformation [6]. When the virus enters host cell, the RBD firstly binds to the angiotensin-converting enzyme 2 (ACE2) receptor on the target cell, triggering a conformational shift of the S protein from the inactive prefusion state to the active postfusion state. The shift leads to the dissociation of the S1 subunit from the S2 subunit, exposing the S2’ cleavage site within the S2 subunit [6]. Subsequently, the S2’ site is cleaved by TMPRSS2 or cathepsin, exposing the FP. Then, HR1 drives FP to insert into the target cell membrane, initiating membrane fusion. Finally, the viral RNA is released into the host cell via fusion pores formed during the merging of viral and cellular membranes [6,15] (Figure 1C). Given the crucial role of the S protein in the viral entry process, it has been identified as a key target for the development of neutralizing antibodies (nAbs), new drugs, and vaccine design.

## 2. Cumulative Mutations in SARS-CoV-2 Spike

SARS-CoV-2 has undergone multiple mutations, resulting in diverse variants. Given the growing risk to global public health, the WHO has classified the SARS-CoV-2 strains as variants of high consequence (VOHCs), variants of concern (VOCs), variants of interest (VOIs), and variants under monitoring (VUMs). Among these, the VOCs, including Alpha (B.1.1.7), Beta (B.1.351), Gamma (P.1), Delta (B.1.617.2), and Omicron (B.1.1.529), are the main drivers of multiple waves of the global COVID-19 pandemic [16]. In late December 2020, Alpha variant was first reported in the United Kingdom. Alpha variant contains 10 amino acid mutations within the NTD, RBD, and furin cleavage sites of the S protein, notably including Δ69-70, Δ144, N501Y, and P681H [8,17]. In the following weeks, the Beta and Gamma variants rapidly spread in South Africa and Brazil, harboring significant mutations in the S protein [8,17]. In May 2021, the Delta variant emerged in India as a VOC, quickly replacing other VOCs and significantly increasing global cases. The Delta variant carries 10 amino acid mutations within the S protein, including T19R, G142D, Δ156-157, R158G located in the NTD, L452R, T478K in the RBD, and P681R in the furin cleavage sites [8,17]. In November 2021, the Omicron variant, first detected in South Africa and Botswana, led to a new global surge in infections and became the dominant strain [17,18]. Over time, Omicron continues to evolve into many different sub-lineages, such as BA.1, BA.2, BA.3, BA.4/5, BF.7, BQ.1, XBB, and EG.5.1, and these new subvariants have shown enhanced ability to transmit and evade antibodies [16,19]. Meanwhile, the WHO has recently identified Omicron sub-lineages XBB.1.5, XBB.1.16, and EG.5 as VOIs. Unlike the Alpha, Beta, Gamma, and Delta variants which are associated with a rise in hospitalization and mortality rates compared to the original strain, the Omicron lineage tends to manifest in a less severe form of the illness [17,20,21]. However, the Omicron variant exhibits over 30 mutations in the S protein, resulting in a significant reduction or even a loss of neutralizing effectiveness for most nAbs [8,16,22,23]. Specifically, studies suggest that the BQ and XBB sub-lineages pose serious threats to the efficacy of existing COVID-19 vaccines, and render all authorized antibodies ineffective [24].

Key mutations and widespread transmission have led to the emergence of viral variants that can evade nAbs, as well as the immunity developed from natural infections and vaccinations. D614G, the first mutation identified in SARS-CoV-2, is universally present across in all VOCs [8]. The emergence and fixation of D614G yielded the variant with enhanced ACE2 binding, transmissibility, and replication, which greatly facilitates virus adaptability [25,26] (Table 1). In the S protein, aside from the D614G mutation, the RBD, NTD, and the furin cleavage site are the primary regions undergoing substantial variations that significantly affect viral behavior [8]. Notably, the Δ69-70 deletion in the NTD, found in diverse variants such as Alpha and Omicron, potentially facilitates viral transmission, but does not primarily contribute to nAbs escape [27,28]. Other mutations in the NTD, including L18F, D80A, H146Y, D253G/Y, and S255F, are associated with reduced or eliminated recognition of NTD-specific antibodies [28]. The N501Y mutation, present in the RBD of Alpha, Beta, Gamma, and Omicron variants, enhances the affinity for ACE2, while simultaneously diminishing the effectiveness of nAbs [29,30]. The variation of the E484 site is observed in the RBD of Beta, Gamma, and Omicron variants. Specifically, both Beta and Gamma variants carry the E484K mutation, which slightly enhances the binding between the RBD and ACE2 [8]. The Omicron variant contains the E484A mutation, which leads to a reduction in binding affinity [8,31,32]. The K417 site variation appears in Beta, Gamma, and Omicron variants. Both Beta and Omicron variants carry the K417N mutation, while Gamma variant displays K417T mutation. The two different substitutions, K417N/T, similarly affect the variants by reducing the affinity between the RBD and ACE2 [33]. However, K417N/T mutations usually co-occur with the N501Y and E484K mutations, which are adequate to compensate for the loss of affinity caused by K417N/T [8]. The L452R mutation, unique to the Delta variant, markedly increases viral infectivity and facilitates immune escape by enhancing affinity for ACE2 [8,34,35] (Table 1). Various single mutations of SARS-CoV-2 variants can impair the efficacy of nAbs that target diverse epitopes [36]. Generally, mutations including K417N, G446S, E484A, and Q493R enable the SARS-CoV-2 variants to escape most class 1 and class 2 antibodies targeting the ACE2 binding site [36,37,38]. Similarly, mutations G339D, N440K, and S371L make several class 3 and class 4 nAbs ineffective [36,38]. Moreover, the P681H mutation, observed within furin cleavage sites in Alpha and Omicron variants, slightly enhances S1/S2 cleavage [39]. The Delta variant, carrying the P681R mutation, significantly facilitates S protein cleavage and cell fusion [40] (Table 1). In summary, the substantial number of mutations in the S protein result in extensive antibody escape, posing a significant challenge to the efficiency of current vaccines and antibody treatments [16,19,41].

## 3. Potent bnAbs against SARS-CoV-2

Antibodies could be generated through natural infection or vaccination. Antibodies produced by natural infection primarily target the N and S proteins, with the vast majority of nAbs targeting the RBD [42,43]. The traditional inactivated virus and live-attenuated virus vaccines present the whole virus to the immune system, thus stimulating the production of antibodies against both the structural and non-structural viral proteins [44]. Studies have shown that the inactivated virus vaccine PiCoVacc can specifically induce the production of nAbs against S, RBD, and N protein in mice, rats, and non-human primates [45]. In contrast, recombinant protein vaccines, vector vaccines, and mRNA vaccines are specifically designed to present certain antigens, such as the S or RBD protein, to produce specific antibodies [44]. For instance, SARS-CoV-2 mRNA vaccines, including Moderna (mRNA-1273) or Pfizer-BioNTech (BNT162b2) encoding full-length S protein, induce functionally diverse antibodies targeting the NTD, RBD, and S2 protein in vaccinees [46,47]. The currently reported nAbs against the SARS-CoV-2 mainly target the S protein, with the majority of potent nAbs focusing on the RBD (Figure 2A). Furthermore, antibodies targeting the NTD and S2 of the S protein have also shown potential in neutralizing emerging SARS-CoV-2 variants. It is critical to continue searching for nAbs that exhibit efficacy against emerging SARS-CoV-2 variants, particularly those with the capacity for broad neutralization against infection of VOCs, including the Omicron sub-lineages. These antibodies, known as broadly neutralizing antibodies (bnAbs), are usually defined as antibodies capable of neutralizing a wide spectrum, even potentially all, circulating virus strains. They usually could achieve this potential by targeting highly conserved epitopes located on the surface proteins of viruses [48].

### 3.1. RBD Antibodies

Antibodies targeting the RBD of SARS-CoV-2 demonstrate significant neutralizing potency; therefore, the majority of antibodies currently undergoing clinical trials are RBD nAbs. These antibodies are further classified based on the specific epitopes they target. In this review, we will primarily adopt the Barnes classification to elucidate these categories [38] (Figure 2B).

#### 3.1.1. Class 1 Antibodies

The class 1 antibodies specifically recognize the “up” conformation of the RBD. The epitope targeted by class 1 antibodies overlaps with the receptor-binding motif (RBM) (Figure 2B,C), allowing these antibodies to effectively neutralize SARS-CoV-2 by blocking the interaction between the RBD and ACE2 [38]. However, prevalent mutations within the RBD, such as K417N, E484K, and N501Y, can weaken the affinity of these antibodies for the RBD, facilitating the viral escape neutralization [49]. Although most class 1 antibodies display limited neutralization breadth across different coronaviruses, a minority of antibodies demonstrate broad and effective neutralizing activity against multiple SARS-CoV-2 variants (Table 2).

Firstly, a series of bnAbs with F486 as a critical central residue have been identified. S2E12, typical of these antibodies, extensively packs F486 within a cavity formed by aromatic residues at the interface between the heavy chain and light chain of the antibody, while mutation residues E484 and S477 are located at the epitope edge. S2E12 exhibits broad neutralizing activity, showing remarkable efficacy against Alpha, Beta, Gamma, Delta, and BA.1, BA.2 variants, with inhibitory concentration (IC_50_) values ranging from 0.6 to 137.2 ng/mL [50,51,52]. The antibodies CB6 (etesevimab) and REGN10933 (casirivimab) also target the F486 site. However, the substitutions in the RBD, including K417N, Q493R substantially decrease activity of CB6 and REGN10933, leading to these antibodies only being able to neutralize Alpha and Delta variants [51,53,54]. Moreover, AZD8895 (tixagevimab), a long-acting IgG molecule of COV2-2196, which forms a hydrogen bond network around the residue F486, effectively neutralizes a range of variants, including VOCs and BA.1 with IC_50_ values ranging from 1.4 to 269.0 ng/mL [51,55,56]. Further structural and functional analysis revealed that antibodies A23-58.1, B1-182.1, Cv2.1169, and 87G7 share a similar RBD recognition pattern with S2E12 [57,58,59]. Both A23-58.1 and B1-182.1 can neutralize the Alpha, Beta, Gamma, Delta, and BA.1 variants with IC_50_ values ranging from 1.6 to 231.0 ng/mL and 0.6 to 281.0 ng/mL, respectively [51,58]. Cv2.1169 and 87G7 are also effective against SARS-CoV-2 VOCs, specifically Omicron sub-lineages BA.1 and BA.2. Notably, Cv2.1169, an IgA antibody generated in mucosal tissues, has been shown to protect mice from Beta infection [57,59]. IgA is the predominant immunoglobulin in the respiratory tract and plays a crucial role in the early prevention of SARS-CoV-2 infection and in limiting the spread of the viruses [60]. Therefore, Cv2.1169 represents a promising candidate for prevention and treatment against COVID-19. Additionally, the nAb 17T2, sharing a high sequence identity with S2E12, engages a larger interaction region with the RBM compared to S2E12. This enhanced area contributes to its high affinity, guaranteeing a complete blockade of the RBD, thereby enabling 17T2 to effectively neutralize a broad spectrum of SARS-CoV-2 variants, including multiple Omicron sub-lineages, BA.1, BA.2, BA.2.86, BA.4/5, BQ.1.1, XBB.1.5, and XBB.1.16 [52]. Importantly, the prophylactic and therapeutic application of 17T2 significantly reduced microscopic lung lesions in a mouse model infected with the Omicron variant [52]. The antibodies exhibit a high degree of resistance against viral escape due to the significant detrimental effects of F486 mutation, which reduce the binding of the RBD to ACE2 and viral replicative fitness [50,61]. However, in subsequent subvariants containing the F486S mutation, such as BA.2.75.2, the neutralizing capacity of these nAbs may be impaired [62].

Another group, the ACE2-mimic antibodies, such as S2K146, with a binding site highly similar to ACE2, effectively block receptor attachment [63]. As a cross-neutralizing antibody, S2K146 shows considerable neutralizing breadth against sarbecoviruses including SARS-CoV-2 variants [63]. Research has confirmed that S2K146 neutralizes VOCs, and maintains effectiveness against the Omicron sub-lineages, including BA.1, BA.1.1, BA.2, BA.3, BA.2.12.1, BA.4/5, BQ.1, and BQ.1.1 [15,63,64]. The antibody P2C-1F11 also shows significant neutralizing effectiveness against VOCs and Omicron sub-lineages including BA.2, BA.2.75, BA.4/5, and BF.7 by mimicking ACE2 and triggering shedding of S1 [65,66]. The epitope of nAbs CT-P59 (regdanvimab) has a substantial overlap with the RBM, exhibiting broad neutralizing activity against VOCs via steric hindrance with ACE2 [67]. Regdanvimab received approval for the treatment of COVID-19 in South Korea in 2021 [68]. Moreover, a group of nAbs, including P2-1B1, P5-1C8, P5S-2B10, P5S-2B6, and P5-1H1, isolated from the peripheral memory B cells, also bind to the RBD by mimicking ACE2 [69]. These antibodies exhibit broad neutralizing capability against a range of SARS-CoV-2 variants, including Alpha, Beta, Gamma, Delta, BA.1, BA.2, BA.2.12.1, BA.2.75, BA.3, and BA.4/5 [69]. GAR05, exhibiting a binding mode similar to that of S2K146, can effectively neutralize BA.1, BA.2, BA.5, and protect K18-hACE2 mice from original SARS-CoV-2 and Delta challenge [70]. Craig et al. reported the isolation of the nAb P4J15 from a convalescent donor, noting its exceptional breadth and neutralization potential against SARS-CoV-2 VOCs, XBB.2.3, and EG.5.1 sub-lineages [71]. Structural analyses revealed that the epitope of P4J15 shares about 93% of its buried surface region with the RBM [71]. In live virus challenge studies, P4J15 offers protection to hamsters against Omicron BA.5 infection and to monkeys against the XBB.1.5 variant [71].

Besides the two types mentioned above, there are also some class 1 bnAbs that cannot be clearly categorized. The antibody 10-5B, isolated from an individual immunized by the inactivated vaccine, demonstrates remarkable efficacy against most SARS-CoV-2 variants, including Omicron subvariants BA.1, BA.2, BA.2.12.1, and BA.3, with IC_50_ values ranging from 2.0 to 46.0 ng/mL [72]. The 10-5B epitope largely overlaps with the RBM site, directly competing with ACE2 for RBD binding [72]. However, the potency of 10-5B significantly diminishes against Omicron BA.4/5 and related subvariants due to mutations S477N, E484A, F486V, which affect the formation of hydrogen bonds between the RBD and 10-5B [72]. Another group of class 1 antibodies, KXD01, KXD02, KXD03, KXD04, KXD05, and KXD06 are cloned from B cells of convalescent donor that probably infected SARS-CoV-2 BA.5 or BF.7 [66]. Remarkably, the antibodies KXD01–06 can broadly neutralize all the VOCs, including Omicron XBB and BQ sub-lineages, and KDX01–03 even show neutralizing effectiveness against the recent EG.5 and FL.1.5 variants [66]. Notably, these nAbs are common found in convalescent individuals, and their considerable neutralization breadth is attributed to extensive affinity maturation [66].

These class 1 antibodies are characterized by their potent and broad neutralizing capability, which enable them effectively to neutralize emerging SARS-CoV-2 variants. These antibodies could be used either alone or in combination with other classes to manufacture therapeutic antibodies and also offer valuable insights for the development of next-generation vaccine design.

#### 3.1.2. Class 2 Antibodies

The class 2 antibodies bind to RBM in both the “up” and “down” RBD, and neutralize by blocking interaction between ACE2 and the RBD, which is similar to class 1 antibodies [38] (Figure 2B,D). Research shows that the K417N and E484K substitutions diminish the binding and neutralization capability of several class 2 antibodies [73]. The therapeutic antibody LY-CoV555 (bamlanivimab) exhibits complete inhibition of binding due to the E484K mutation [74]. Furthermore, this mutation also decreases the binding affinity of P2B-2F6 to the S protein, thereby restricting the neutralization capability of LY-CoV555 and P2B-2F6 to only the Alpha VOC [75,76,77]. In contrast, another antibody, MD65, showing a similar binding profile to LY-CoV555, is not impacted in its binding efficacy by the E484K mutation [78]. MD65 exhibits efficacy against Alpha, Beta, and Gamma variants in an in vivo assessment of K18-hACE2 transgenic mice [78]. Additionally, P5S-2A9, isolated from peripheral memory B cells, displays broad neutralizing efficacy against multiple VOCs, including BA.2, BA.2.12.1, BA.2.75, BA.3, and BA.4/5 [69] (Table 2). Remarkably, although P5S-2A9 exhibits low neutralization potency, such broad neutralization is rare among class 2 antibodies, suggesting the potential for developing broadly reactive class 2 nAbs [69].

#### 3.1.3. Class 3 Antibodies

The class 3 antibodies recognize non-RBM epitopes on the RBD in both “up” and “down” conformation [38] (Figure 2B,E). The majority of class 3 antibodies target a relatively conserved and buried region, maintaining considerable neutralizing activity against Omicron sub-lineages [79]. However, the emergence of XBB, BQ.1, and subsequent variants has led to the loss of conservation in this region, likely attributed to new mutations including R346T, K444T, V445P, and N460K, which have been identified as key factors in severe immune escape [79,80].

The typical class 3 nAb, S309 (sotrovimab), specifically binds to an epitope containing a glycan at the N343 site universally conserved across the sarbecovirus, without blocking ACE2 attachment [81]. Identified in a patient infected with SARS-CoV in 2003 and subsequently isolated in 2013, S309 exhibits a strong affinity for the SARS-CoV-2 RBD [81]. Additionally, it demonstrates broad neutralization against SARS-CoV-2 VOCs, and Omicron subvariants BA.1.1, BA.2, BA.2.12.1, BA.2.75, BA.4/5, XBB [79]. In May 2021, sotrovimab, a stabilized derivative of S309 optimized for enhanced Fc receptor affinity, was granted emergency use authorization as a therapeutic antibody to prevent disease progression in high-risk, early-stage COVID-19 patients [82,83]. Although S309 has a high neutralizing breadth, its neutralizing efficacy against several variants is relatively low. Particularly, the mutations R346T, K444T, and V445P in the RBD notably increase immune evasion in the BF.7, BQ, and XBB sub-lineages [79]. The antibody SP1-77 interacts with the N343 glycosylation site in a manner similar to S309 and exhibits a superior neutralizing effectiveness against VOCs, BA.1, BA.2, BA.3, BA.4/5, and BA.2.12.1 variants, with IC_50_ values ranging from 6.5 to 76.0 ng/mL [84]. Unlike class 1 and class 2 antibodies, S309 and SP1-77 do not inhibit attachment between SARS-CoV-2 and the host cell. Instead, S309 blocks the virus through mechanisms like S trimer cross-linking, steric hindrance, or virion aggregation, and SP1-77 prevents fusion of viral and cellular membranes by blocking S1 dissociation, impacting the subsequent steps of the SARS-CoV-2 infection process [81,84]. The antibody SW186 recognizes a conserved epitope outside the RBM, allowing it to maintain neutralizing effectiveness against various SARS-CoV-2 variants, such as Alpha, Beta, Gamma, Delta, and BA.1 [85]. The SW186 epitope consists of conserved amino acids, particularly the N343 glycosylation site, which plays a pivotal role in the transition of the RBD from a glycan-shielded “down” conformation to an accessible “up” conformation, crucial for viral entry into host cells. Although SW186 does not directly bind to the RBM, it still exhibits partial inhibition of the binding interaction between the RBD and ACE2 [85].

Another antibody, LY-CoV1404 (bebtelovimab), isolated from a convalescent donor, demonstrates strong neutralization capability for prevalent VOCs, and Omicron subvariants BA.1, BA.2, BA.2.12.1, BA.2.75, BA.4/5, BF.7 with IC_50_ values between 0.7 and 2.2 ng/mL [79,86]. The LY-CoV1404 targets a region accessible in both the “up” and “down” conformations of the RBD, partially overlapping with the RBM [86]. Consequently, LY-CoV1404 impedes the binding of ACE2, explaining the strong mechanism for its potent neutralizing activity [86]. Although this property suggests that LY-CoV1404 is a class 2 antibody, the location of its epitope is closer to the canonical class 3 antibody S309 [86]. Further analysis demonstrates that aside from the common mutations N439 and N501, the contact residues of the LY-CoV1404 epitope are highly conserved. However, the binding and neutralizing capability of LY-CoV1404 remains unaffected by N439K and N501Y mutations [86]. AZD1061 (cilgavimab), a long-acting IgG molecule of COV2-2130, targeting the RBD and partially overlapping with the RBM, shows effective neutralization against VOCs and BA.1. The antibody cocktail AZD7442, incorporating AZD8895 and AZD1061, has been used for patient treatment of COVID-19 [55]. S2X324 recognizes a similar region to LY-CoV1404, which partially overlaps with the RBM [87]. S2X324 exhibits cross-reactivity and effectively neutralizes Omicron subvariants BA.1, BA.2, BA.3, BA.4, BA.5, BA.2.12.1, and BA.2.75 with IC_50_ values below 10.0 ng/mL, except BA.2.75, with IC_50_ value 18.0 ng/mL [87]. Biochemical experiments reveal that S2X324 primarily inhibits SARS-CoV-2 infection by blocking ACE2 binding [87]. Additionally, the antibody P2S-2E9, with extensive overlap in binding footprints with LY-CoV1404, effectively neutralizes various SARS-CoV-2 variants, including VOCs, and Omicron subvariants BA.1, BA.2, BA.2.12.1, BA.2.75, BA.3, and BA.4/5 [69].

There are also some antibodies which target epitopes outside the N343 glycan site and the RBM, thereby positioning them uniquely between the binding domains of S309 and LY-CoV1404 [79]. REGN10987 (imdevimab) is one of these antibodies, with its epitope located on the side of the RBD and little to no overlap with the RBM, showing neutralizing activity against Alpha, Beta, Gamma and Delta [51,54]. Similarly, the antibody 1G11 demonstrates robust neutralizing activity against pseudotyped SARS-CoV-2 VOCs, maintaining a high level against Omicron subvariants BA.1, BA.1.1, BA.2, BA.2.12.1, BA.4/5, and BF.7 [79]. The antibody 1G11 does not interfere with binding of the RBD with ACE2; instead, it promotes cross-linking of spikes after binding to the RBD, clarifying its mechanism for potent ACE2 blockade and exceptional neutralization efficacy [79]. Moreover, 002-S21F2 recognizes a conformationally conserved epitope located on the external surface of the RBD. It has shown consistent neutralizing efficacy against a range of SARS-CoV-2 variants, including VOCs, BA.1, BA.2, BA.2.12.1, BA.4, and BA.5, with IC_50_ values between 20.0 and 130.0 ng/mL [88]. Additionally, the antibody 6-2C, derived from individual immunized with the inactivated vaccine, can broadly neutralize VOCs, and the variants highly evasive to antibodies, such as XBB.1.5, XBB, BQ.1.1, and BA.2.75.2 [72] (Table 2). The epitope targeted by 6-2C is mutationally constrained in terms of folding and expression, which accounts for its broad neutralization potential [72].

#### 3.1.4. Class 4 Antibodies

The class 4 antibodies bind to non-RBM epitopes in the “up” RBD [38,89] (Figure 2B,F). The majority of antibodies previously described are cross-reactive but weakly neutralizing [90,91].

The classic class 4 antibody CR3022, isolated from a convalescent SARS patient, specifically recognizes a highly conserved epitope shared between SARS-CoV-2 and SARS-CoV, distinct from the RBM [89]. Further structural analysis demonstrates that the CR3022 epitope is accessible only when at least two RBDs of the S trimer are in “up” conformation with a slight rotation [89]. The epitope of CR3022 is inaccessible in the prefusion state of the spike, suggesting that its binding facilitates transition to the postfusion state [92]. Cryogenic electron microscopy analysis confirms that incubation of CR3022 Fab with spike protein results in disruption of the prefusion trimer [92]. Despite the emergence of multiple VOCs and Omicron sub-lineages, only one residue mutation within the CR3022 epitope is observed, underlining its potential in therapeutic applications [93]. However, antibodies targeting the CR3022 epitope display a broad binding breadth but low neutralization strength against SARS-CoV-2 [90]. Specifically, COVA1-16, which also targets this epitope, loses its neutralization activity against the Omicron variant [94]. The diminished effectiveness can be attributed to multiple factors: insufficient affinity, the relative inaccessibility of the epitope, or a direct competition failure with the ACE2 receptor [90]. Notably, the nAb ADG-20, an affinity-matured derivative of ADI-55688, interacting with the CR3022 epitope, demonstrates neutralizing efficacy against BA.1 and BA.1.1 by competing with ACE2 [90].

Additionally, some class 4 antibodies exhibit better neutralizing activity; these antibodies recognize epitopes different from those of CR3022. The nAb S2X259, derived from the memory B cells of a COVID-19 convalescent donor, recognizes a cryptic conserved epitope of the RBD and demonstrates cross-reactivity with spike proteins of all sarbecovirus [91]. It can broadly neutralize SARS-CoV-2 VOCs, Omicron subvariants BA.1, BA.1.1, BA.3, and a range of sarbecoviruses, by inhibiting ACE2 binding [19,91]. Remarkably, the S2X259 epitope is conserved across currently circulating strains and does not comprise prevalent RBD mutations, such as S477N, N439K or L452R [91]. Further analyses demonstrate that the rare G504D substitution is the only escape mutation for S2X259, suggesting this nAb might have a high barrier against the SARS-CoV-2 escape mutants [91]. Another cross-reactive antibody, DH1047, has been proven in its efficacy in neutralizing BA.1, BA.1.1 by targeting a conserved epitope within the RBD [19,95]. Similarly, the nAb 10-40 exhibits broad neutralizing capability against a range of variants of SARS-CoV-2, including Omicron subvariants BA.1, BA.1.1, BA.2, BA.2.12.1, BA.4/5, and also SARS-CoV [19,96] (Table 2). The epitopes of 10-40 and DH1047 significantly overlap; however, the angles at which they approach the targeted site are different, highlighting that the targeted site is a viable target for developing a universal sarbecovirus vaccine [96].

The class 3 and class 4 epitopes exhibit a high level of conservation across sarbecoviruses, suggesting their potential as effective targets for bnAbs, and indicating that the epitopes maintain functional stability and have a reduced association with immune escape mechanisms [38]. Additionally, class 3 and class 4 antibodies enrich the SARS-CoV-2 antibody repertoire, probably offering effective use in therapeutic combinations with class 1 and class 2 nAbs, which lead to additive neutralization effects and simultaneously curb viral escape mechanisms.

#### 3.1.5. Class 5 Antibodies

The class 5 antibodies target a cryptic region, previously described as the “E465 patch”, that is highly conserved among diverse sarbecoviruses, including SARS-CoV-2 [31,61]. The class 5 epitope substantially overlaps with the NTD-interacting region and is exposed only when the spike protein adopts the “up” conformation with at least one RBD accessible [61,97] (Figure 2B,G).

The antibody S2H97 exhibits high-affinity binding to a cryptic conserved epitope across all sarbecovirus clades, which is designated as site 5 (Figure 2G). This binding requires a substantial opening of the RBD to expose its specific epitope, accelerating the transmission of spike protein to the postfusion conformation and effectively inhibiting viral entry. S2H97 effectively neutralizes diverse sarbecoviruses and SARS-CoV-2 variants, including Omicron BA.1 [61]. The antibody XMA09 exhibits high epitope similarity with S2H97 and maintains comparable neutralizing capability against the Omicron pseudovirus to that of D614G [98]. ION_300 binds to a distinctive region on the opposite side of the RBM, obscured by the NTD of an adjacent S1 polypeptide chain when the RBD is in the closed conformation [99]. Analysis of epitope sites predicts that this antibody is expected to maintain binding and neutralization potency against prevalent RBD mutations, such as K417N/T, E484K, and N501Y [99]. The nAbs WRAIR-2057 and WRAIR-2063, isolated from a convalescent donor, can potently neutralize SARS-CoV and SARS-CoV-2. Moreover, they retain neutralization against various Omicron subvariants, including BA.1, BA.2, BQ.1.1, and XBB.1.5 [93,100]. The epitope of WRAIR-2057 exhibits 48% overlap with S2H97 and 70% with ION_300 [93]. The epitope of WRAIR-2063 is accessible only when one or more RBDs is in the “up” conformation, which may facilitate the shift of spike protein from prefusion to postfusion, resulting in the rapid disintegration of the S trimer [97,101]. The superimposition of the WRAIR-2063–RBD complex onto the RBD of the S trimer reveals no significant steric clashes with RBDs of the adjacent protomers, suggesting that multiple WRAIR-2063 antibodies could bind to the S trimer [97]. The antibodies FD20, 7D6, and 6D6 show a similar neutralization mechanism to that of WRAIR-2063, which involves disassembling the spike protein [97,101]. FD20, isolated from a COVID-19 convalescent patient, can neutralizes Alpha, Beta, Gamma, and Delta [97,101]. The murine cross-neutralizing antibodies 7D6 and 6D6 showed almost consistent neutralizing efficacy against Alpha, Beta, and Gamma variants. The binding of 7D6 and 6D6 with the RBD causes steric hindrance with the adjacent NTD, thereby disrupting the viral spike [97,101,102]. Furthermore, research demonstrates that the interaction of the S protein with CR3022 and other antibodies recognizing cryptic epitopes is notably improved when WRAIR-2063 is present, suggesting that engagement with WRAIR-2063 induces conformational alterations in the S trimer, thereby affecting accessibility of these cryptic sites [97]. This may indicate that binding of class 5 antibodies leads to more significant conformational rearrangements in the S trimer, ultimately impacting neutralization effectiveness and breadth of antibodies that aim at associated epitopes.

Despite the continuous emergence of VOCs including Omicron, none has yet revealed mutations within the class 5 epitope, highlighting the therapeutic potential of these antibodies [93]. The class 5 antibodies exhibit a spectrum of neutralization capability and a considerable breadth against a variety of sarbecoviruses (Table 2), emphasizing their critical role in the development of vaccine and therapeutics.

### 3.2. NTD Antibodies

The limited immunogenicity of the SARS-CoV-2 NTD in COVID-19 patients has been attributed to its extensive N-linked glycan shielding [103,104]. However, recent studies report the isolation of NTD antibodies that show neutralizing capability against SARS-CoV-2 infection in vitro, indicating their potential utility in COVID-19 prophylaxis or treatment [28] (Table 2). The first nAb targeting the SARS-CoV-2 NTD, 4A8, was reported by Chi et al., and exhibits neutralizing potency with an IC_50_ value of 0.39 μg/mL against the authentic SARS-CoV-2 virus [105]. Matthew et al. described an antigenic map of the heavily glycosylated SARS-CoV-2 NTD by characterizing 41 NTD human antibodies, in which six antigenic sites (i–vi) were designated [28]. The most potent nAbs, including 4A8, competed for binding to the NTD site i, which is identified as a supersite. The NTD is highly glycosylated, and its supersite, positioned at the periphery of the spike protein, represents the largest glycan-free area on the NTD [106]. The three regions, including the NTD N-terminal region, residues 14–20, a β-hairpin formed by residues 140–158, and a loop spanning residues 245–264, targeted by antibodies S2L28, S2M28, and S2X333, together constitute an antigenic supersite at the apex of the NTD, on the side distal to the viral membrane. Among these, S2X333 inhibits the fusion between cells, activates effector functions, and offers protection to hamsters against SARS-CoV-2 challenge [28]. However, SARS-CoV-2 VOCs possess a large number of mutations within the NTD supersite, potentially constraining the recognition and neutralizing capability of nAbs at this supersite [28,107,108]. The antibody 4–18, targeting the supersite of the NTD, loses neutralizing activity against all Omicron subvariants [106,109]. Another potent nAb 5–7, recognizing the NTD outside of the supersite, retains neutralization potency against VOCs, including BA.1 and BA.1.1, with a binding site remote from most mutations [19,110]. In general, nAbs targeting the NTD achieve neutralization by locking the S protein thereby inhibiting conformational transitions to postfusion state, rather than by directly blocking the ACE2 receptor [111].

Generally, the NTD region exhibits significant variability across various coronaviruses, making it an unlikely target for cross-neutralizing antibodies. Nevertheless, combining NTD nAbs with RBD antibodies may enhance the efficacy of antibody cocktails.

### 3.3. S2 Antibodies

Compared to the S1 subunit, the amino acid sequences of the S2 subunit are relatively conserved, which is consistent with its conserved function [112]. The S2 subunit plays an important role in facilitating the fusion of viruses with host cell membranes, suggesting a low tolerance for variants without affecting viral fitness. Studies have found that S2 contains immunodominant epitopes capable of eliciting nAbs [113,114]. Despite the limited production of S2-specific antibodies following natural SARS-CoV-2 infection or vaccination, and only a few antibodies showing neutralizing activity, those targeting specific S2 epitopes tend to show broad neutralizing potential [115,116,117] (Table 2).

Notably, the S2 stem helix region within residues 1141–1160 of the S protein demonstrates conservation across various SARS-CoV-2 VOCs [118]. Therefore, it represents an optimal target for the development of bnAbs. The S2 stem helix-specific antibody 1249A8 can universally recognize all human β-coronaviruses and effectively neutralize SARS-CoV-2, SARS-CoV, and MERS-CoV by inhibiting the post-attachment fusion process. Furthermore, it also shows prophylactic activities against SARS-CoV-2 WA-1, Beta, and Omicron strains in K18 hACE2 mice [117]. Competition surface plasmon resonance (SPR) results reveal that human nAbs CV3-25, CC40.8, and S2P6 target the epitope overlapping with that of 1249A8. They can neutralize various SARS-CoV-2 VOCs by interfering the membrane fusion crucial for viral infection [119,120,121,122,123]. However, only CV3-25 is capable of neutralizing the Omicron variant, possibly because its binding epitope extends more towards the C-terminal [122,123]. The antibody WS6, isolated from mice immunized with mRNA encoding the S protein of SARS-CoV-2, could neutralize Alph, Beta, Gamma, Delta, Omicron, and SARS-CoV, by inhibiting the conformational changes of the S protein [120]. Similarly, a group of murine antibodies, S2-4A, S2-4D, S2-5D, and S2-8D, also exhibit broad neutralizing activity against these VOCs [124]. Furthermore, the FP sequence within the S2 subunit is completely conserved across all SARS-CoV-2 VOCs. The FP-specific antibody 76E1, derived from a COVID-19 convalescent donor, effectively neutralizes the SARS-CoV-2 VOCs, exhibiting no residue mutations in the 76E1 epitope across current SARS-CoV-2 VOCs [125]. Structural analysis revealed that the epitope of 76E1 is buried within the prefusion S trimer, and a conformational change from the prefusion to the postfusion state leads to the exposure of the epitope to allow binding of 76E1 [125]. Then, 76E1 suppresses membrane fusion by blocking the cleavage of S2’ [125]. The binding of ACE2 facilitates the exposure of the S2’ site and the fusion peptide, hence 76E1 has a synergistic effect with ACE2 in preventing SARS-CoV-2 infection, suggesting that a potential combination of 76E1 with ACE2 or RBD antibodies could prevent SARS-CoV-2 infection. The antibodies VN01H1 and C77G12 can neutralize the Omicron subvariants BA.1 and BA.2 [126]. Similar to 76E1, their binding sites are located within the prefusion S trimer, which allows them to also work synergistically with ACE2 or ACE2-mimicking antibodies to enhance the neutralizing effect [126]. The other two FP-specific antibodies, COV44-62 and COV44-79, demonstrate a broad capacity to neutralize SARS-CoV-2 VOCs, and Omicron subvariants BA.1, BA.2, BA.4/5, by inhibiting virus and host membrane fusion [127]. The epitope mapping reveals that COV44-62 and COV44-79 bind to the same region of the FP helix from distinct angles, with part of their epitope accessible in the prefusion spike protein [127]. Furthermore, binding analysis shows that COV44-62 and COV44-79 exhibit higher affinity for the S2 subunit compared to the prefusion S protein, suggesting that their binding epitopes might be buried by the S1 subunit. This might also explain why FP-specific antibodies are only produced in a subset of COVID-19 patients and rarely during natural infection or vaccination [127]. hMab5.17, a humanized antibody, targets the HR2 region and is capable of effectively neutralizing both SARS-CoV and SARS-CoV-2 VOCs [128]. Given that the HR2 region exhibits nearly 100% conservation across SARS-CoV-2 VOCs, it suggests a potential for hMab5.17 to elicit bnAbs against SARS-CoV-2 [118].

Although antibodies targeting the S2 subunit display broadly neutralizing activity, they tend to exhibit lower neutralizing titers compared to those targeting the RBD [118]. Therefore, enhancing the potency of antibodies specific to the S2 subunit remains a significant challenge. Moreover, combination of S1 and S2 antibodies may represent a promising strategy for COVID-19 treatment, potentially preventing the emergence of immune escape strains.

### 3.4. Other Antibodies

The single-domain antibodies (sdAbs), derived from the heavy chains of camelid or cartilaginous fish immunoglobulins, termed nanobodies, present a compelling alternative to mAbs [129]. These nanobodies are the smallest naturally occurring antigen-binding domains (~15 kDa), offering multiple advantages over conventional mAbs, including better epitope accessibility, superior solubility and stability, easier production, and lower costs [130]. Importantly, nanobodies can be administered by inhaled delivery because of their deep tissue penetration, making them ideally suited for treatment of respiratory diseases [131]. Nanobodies based on camelids are one of the prominent candidates for viral nAbs. Li et al. reported several nanobodies targeting the RBD of SARS-CoV-2 from immunized alpacas, including Nb70, 1-2C7, and 3-2A2-4, exhibiting effectiveness against various SARS-CoV-2 VOCs and Omicron subvariants BA.1, BA.2, BA.4/5, and SARS-CoV [132]. Notably, nanobody 3-2A2-4 specifically binds to a conserved epitope on the bottom of the RBD between the cryptic and the outer face and protects K18-hACE2 mice from Omicron and Delta infection [132]. In addition, nanobodies from sharks might have variable new antigen receptors. The novel nanobody S2A9, from a naive nurse shark, targeting the S2 stem helix region, has shown broad neutralization capability against pseudotyped SARS-CoV-2 VOCs and Omicron subvariants BA.1, BA.2, BA.4/5 [133]. Furthermore, engineering nanobodies into multivalent forms might improve their efficacy and stability. Specifically, the creation of the trivalent and bispecific Nb_15_-Nb_H_-Nb_15_ from an alpaca offers protection against SARS-CoV-2 infection in transgenic hACE2 mice for both preventive and therapeutic uses [134]. Although nanobodies possess unique properties, the lack of an Fc fragment limits their ability to recruit the functions of immune system effectors. Therefore, overcoming this limitation by molecular modification or coupling with other molecules, simultaneously, prolonging half-life and reducing immunogenicity of nanobodies in vivo will further expand their therapeutic potential. Generally, the development of nanobodies will help to accelerate research on bnAbs, crucial for the prevention and treatment of viral outspreads.

## 4. Conclusions

The emerging SARS-CoV-2 variants constantly escape neutralization provided by antibodies from current vaccines and natural infections. Upon the advent of the BQ.1, BQ.1.1, and XBB.1 lineages, all the approved therapeutic antibodies have completely lost their effectiveness [135]. Moreover, under the selective immune pressure resulting from the widespread prevalence of viruses circulating in the general population, SARS-CoV-2 seems likely to continue. Therefore, it is necessary to develop new antibodies to combat the currently circulating variants and prepare for future strains. Only a few antibodies targeting the RBD, NTD, and S2 have shown broad and potent efficacy against SARS-CoV-2 VOCs, including Omicron. Analysis of antigen epitopes reveals that the class 1 and class 2 nAbs that target the RBD directly compete with ACE2 for binding to the RBM, often demonstrating the most potent neutralization activity. However, the RBM is noted for its poor conservation and susceptibility to mutations, which restricts the effectiveness of class 1 and class 2 nAbs against SARS-CoV-2 variants [136]. In contrast, the class 3 and class 4 nAbs, which target areas distal to the RBM, typically show lower neutralization potency but are more conserved compared to the class 1 and class 2 nAbs [136]. To date, class 5 nAbs have been consistently reported to have broad and moderate neutralization potential, with almost entirely conserved epitopes [97]. The neutralizing activities of nAbs targeting the NTD and S2 sites are generally significantly lower than that of nAbs targeting the RBD [137]. Combining these neutralizing antibodies targeting different epitopes or designing bispecific antibodies for cocktail therapy are promising treatment options for patients with severe COVID-19. Specifically, antibody therapy offers potential clinical benefits for individuals at high risk with compromised immune functions, as they are unable to generate a protective humoral immune response following vaccination [138,139]. Furthermore, vaccines specifically targeting the epitopes of these broadly neutralizing antibodies may suggest an excellent strategy for the preparedness to curb next SARS-CoV-2 pandemic.

## Figures and Tables

**Table 1:** Key mutations in the S protein of VOCs and their impacts [8].

Site of the Mutations	Mutations	Mutations Shared with VOCs	Impact of the Mutation
NTD	Δ69-70	Alpha, Omicron	decreases neutralization potency
	T95I	Omicron	increases transmission, associated with immune escape
RBD	G339D	Omicron	increases transmission by enhancing interaction between S protein and ACE2
	S371L	Omicron	increases immune escape
	K417N/T	Beta, Gamma, Omicron	reduces affinity between the RBD and ACE2, associated with immune escape
	N440K	Omicron	increases binding between the RBD and ACE2
	L452R	Delta	increases affinity between the RBD and ACE2, promotes immune escape
	T478K	Delta, Omicron	enhances the ACE2 interaction, increases immune escape
	E484A/K	Beta, Gamma, Omicron	E484K enhances binding between the RBD and ACE2, significantly increases immune escape E484A decreases affinity between the RBD and ACE2, stimulates severe immune escape
	Q493R	Omicron	contributes to immune escape
	N501Y	Alpha, Beta, Gamma, Omicron	increases transmission by enhancing interaction between S protein and ACE2
S1/S2 cleavage site	D614G	Alpha, Beta, Gamma, Delta, Omicron	increases transmission
	P681H/R	Delta, Omicron	facilitates S cleavage, endows moderate immune escape ability

**Table 2:** Neutralizing antibodies with potent neutralizing effectiveness against SARS-CoV-2 variants.

Target	Epitope	NAbs	Origin	SARS-CoV-2 VOCs and Omicron Sub-Lineages	Emergency Use Authorization
RBD	Class 1	S2E12	Human	Alpha, Beta, Gamma, Delta, BA.1, BA.2	
		A23-58.1	Human	Alpha, Beta, Gamma, Delta, BA.1	
		B1-182.1	Human	Alpha, Beta, Gamma, Delta, BA.1	
		Cv2.1169	Human	Alpha, Beta, Gamma, Delta, BA.1, BA.2	
		87G7	Human	Alpha, Beta, Gamma, Delta, BA.1, BA.2	
		17T2	Human	Alpha, Beta, Gamma, Delta, BA.1, BA.2, BA.4/5, BQ.1.1, XBB.1.5, XBB.1.16, BA.2.86	
		CB6	Human	Alpha, Delta	Etesevimab *
		REGN10933	Human	Alpha, Delta	Casirivimab *
		AZD8895	Human	Alpha, Beta, Gamma, Delta, BA.1	Tixagevimab *
		S2K146	Human	Alpha, Beta, Gamma, Delta, BA.1, BA.1.1, BA.2, BA.3, BA.2.12.1, BA.4/5, BQ.1, BQ.1.1	
		GAR05	Human	Alpha, Beta, Gamma, Delta, BA.1, BA.2, BA.5	
		P2C-1F11	Human	Alpha, Beta, Gamma, Delta, BA.2, BA.2.75, BA.4/5, BF.7	
		CT-P59	Human	Alpha, Beta, Gamma, Delta, Omicron	Regdanvimab
		P4J15	Human	Alpha, Beta, Gamma, Delta, BA.1, BA.4/5, BA.2.75.2, BQ.1, BQ.1.1, XBB.1, XBB.1.5, CH.1.1, XBB.1.16, XBB.1.16.1, XBB.2.3, EG.1, EG.5.1	
		P2-1B1	Human	Alpha, Beta, Gamma, Delta, BA.1, BA.2, BA.2.12.1, BA.2.75, BA.3, BA.4/5	
		P5-1C8	Human	Alpha, Beta, Gamma, Delta, BA.1, BA.2, BA.2.12.1, BA.2.75, BA.3, BA.4/5	
		P5S-2B10	Human	Alpha, Beta, Gamma, Delta, BA.1, BA.2, BA.2.12.1, BA.2.75, BA.3, BA.4/5	
		P5S-2B6	Human	Alpha, Beta, Gamma, Delta, BA.1, BA.2, BA.2.12.1, BA.2.75, BA.3, BA.4/5	
		P5-1H1	Human	Alpha, Beta, Gamma, Delta, BA.1, BA.2, BA.2.12.1, BA.2.75, BA.3, BA.4/5	
		10-5B	Human	Alpha, Beta, Gamma, Delta, BA.1, BA.2, BA.2.12.1, BA.3	
		KXD01	Human	Alpha, Beta, Gamma, Delta, BA.1, BA.2, BA.3, BA.4/5, BA.2.75, BF.7, BQ.1, XBB, XBB.1, XBB.1.5, XBB.1.16, EG.5, EG.5.1, FL.1.5, FL.1.5.1	
		KXD02	Human	Alpha, Beta, Gamma, Delta, BA.1, BA.2, BA.3, BA.4/5, BA.2.75, BF.7, BQ.1, XBB, XBB.1, XBB.1.5, XBB.1.16, EG.5, EG.5.1, FL.1.5, FL.1.5.1	
		KXD03	Human	Alpha, Beta, Gamma, Delta, BA.1, BA.2, BA.3, BA.4/5, BA.2.75, BF.7, BQ.1, XBB, XBB.1, XBB.1.5, XBB.1.16, EG.5, EG.5.1, FL.1.5, FL.1.5.1, HK.3	
		KXD04	Human	Alpha, Beta, Gamma, Delta, BA.1, BA.2, BA.3, BA.4/5, BF.7, BQ.1, XBB.1.5, XBB.1.16	
		KXD05	Human	Alpha, Beta, Gamma, Delta, BA.1, BA.2, BA.3, BA.4/5, BA.2.75, BF.7, BQ.1, XBB.1.5	
		KXD06	Human	Alpha, Beta, Gamma, Delta, BA.1, BA.2, BA.3, BA.4/5, BA.2.75, BF.7, BQ.1, XBB.1.5, XBB.1.16	
	Class 2	LY-CoV555	Human	Alpha	Bamlanivimab *
		P2B-2F6	Human	Alpha	
		MD65	Human	Alpha, Beta	
		P5S-2A9	Human	Alpha, Beta, Gamma, Delta, BA.2, BA.2.12.1, BA.2.75, BA.3, and BA.4/5	
	Class 3	S309	Human	Alpha, Beta, Gamma, Delta, BA.1.1, BA.2, BA.2.12.1, BA.2.75, BA.4/5, XBB	Sotrovimab *
		SP1-77	Humanised Mouse	Alpha, Beta, Gamma, Delta, BA.1, BA.2, BA.3, BA.4/5, and BA.2.12.1	
		SW186	Mouse	Alpha, Beta, Gamma, Delta, BA.1	
		LY-CoV1404	Human	Alpha, Beta, Gamma, Delta, BA.1, BA.2, BA.2.12.1, BA.2.75, BA.4/5, BF.7	Bebtelovimab *
		AZD1061	Human	Alpha, Beta, Gamma, Delta	Cilgavimab *
		S2X324	Human	Alpha, Beta, Gamma, Delta, BA.1, BA.2, BA.3, BA.4, BA.5, BA.2.12.1, BA.2.75	
		P2S-2E9	Human	Alpha, Beta, Gamma, Delta, BA.1, BA.2, BA.2.12.1, BA.2.75, BA.3, BA.4/5	
		REGN10987	Human	Alpha, Beta, Gamma, Delta	Imdevimab *
		1G11	Human	Alpha, Beta, Gamma, Delta, BA.1, BA.1.1, BA.2, BA.2.12.1, BA.4/5, BF.7	
		002-S21F2	Human	Alpha, Beta, Gamma, Delta, BA.1, BA.2, BA.2.12.1, BA.4/5	
		6-2C	Human	Alpha, Beta, Gamma, Delta, BA.1, BA.2.12.1, BA.2.75, BA.3, BA.4/5, BA.4.6, BF.7	
		3-2A2-4	Alpaca	Alpha, Beta, Gamma, Delta, BA.1, BA.2, BA.4/5	
	Class 4	COVA1-16	Human	Alpha, Beta, Gamma, Delta	
		ADG-20	Human	Alpha, Beta, Gamma, Delta, BA.1, BA.1.1	
		S2X259	Human	Alpha, Beta, Gamma, Delta, BA.1, BA.1.1, BA.3	
		DH1047	Human	Alpha, Beta, Gamma, Delta, BA.1, BA.1.1	
		10-40	Human	Alpha, Beta, Gamma, Delta, BA.1, BA.1.1, BA.2, BA.2.12.1, and BA.4/5	
		Nb70	Alpaca	Alpha, Beta, Gamma, Delta, BA.1, BA.2, BA.4/5	
		1-2C7	Alpaca	Alpha, Beta, Gamma, Delta, BA.1, BA.2, BA.4/5	
	Class 5	S2H97	Human	Alpha, Beta, Gamma, Delta, BA.1	
		XMA09	Human	Alpha, Beta, Gamma, Delta, Omicron	
		WRAIR-2057	Human	Alpha, Beta, Gamma, Delta, BA.1, BA.2, BQ.1.1, XBB.1.5	
		WRAIR-2063	Human	Alpha, Beta, Gamma, Delta, BA.1, BA.2, BQ.1.1, XBB.1.5	
		FD20	Human	Alpha, Beta, Gamma, Delta	
		7D6	Mouse	Alpha, Beta, Gamma	
		6D6	Mouse	Alpha, Beta, Gamma	
S2	stem helix	1249A8	Human	Alpha, Beta, Gamma, Delta, Omicron	
		CV3-25	Human	Alpha, Beta, Gamma, Delta, Omicron	
		CC40.8	Human	Alpha, Beta, Gamma, Delta	
		S2P6	Human	Alpha, Beta, Gamma, Delta	
		WS6	Mouse	Alpha, Beta, Gamma, Delta, Omicron	
		S2-4D	Mouse	Alpha, Beta, Gamma, Delta, Omicron	
		S2-5D	Mouse	Alpha, Beta, Gamma, Delta, Omicron	
		S2-8D	Mouse	Alpha, Beta, Gamma, Delta, Omicron	
		S2-4A	Mouse	Alpha, Beta, Gamma, Delta, Omicron	
		S2A9	Shark	Alpha, Beta, Gamma, Delta, BA.1, BA.2, BA.4/5	
	fusion peptide	76E1	Human	Alpha, Beta, Gamma, Delta, Omicron	
		VN01H1	Human	Alpha, Beta, Gamma, Delta, BA.1, BA.2	
		C77G12	Human	Alpha, Beta, Gamma, Delta, BA.1, BA.2	
		COV44-62	Human	Alpha, Beta, Gamma, Delta, BA.1, BA.2, BA.4/5	
		COV44-79	Human	Alpha, Beta, Gamma, Delta, BA.1, BA.2, BA.4/5	
	heptad repeat	hMab5.17	Humanised from Immunised Mouse	Alpha, Beta, Gamma, Delta	
NTD		4-18	Human	Alpha, Beta, Gamma, Delta	
		5-7	Human	Alpha, Beta, Gamma, Delta, BA.1, BA.1.1	

* Due to the high frequency of circulating SARS-CoV-2 variants, the antibody is not a currently authorized treatment of COVID-19 under the Emergency Use Authorization until further notice by the Agency.

## Data Availability

All relevant data are within the manuscript.
